# Microbial Fuels Cell-Based Biosensor for Toxicity Detection: A Review

**DOI:** 10.3390/s17102230

**Published:** 2017-09-28

**Authors:** Tuoyu Zhou, Huawen Han, Pu Liu, Jian Xiong, Fake Tian, Xiangkai Li

**Affiliations:** 1 Ministry of Education, Key Laboratory of Cell Activities and Stress Adaptations, School of Life Science, Lanzhou University, Tianshui South Road #222, Lanzhou 730000, China; zhouty2016@lzu.edu.cn (T.Z.); hanhw13@lzu.edu.cn (H.H.); 2 Department of Development Biology Sciences, School of Life Science, Lanzhou University, Tianshui South Road #222, Lanzhou 730000, China; liupu@lzu.edu.cn; 3 Wuhan Optics Valley Bluefire New Energy Co., Ltd., Three Hubei Road, Wuhan East Lake Development Zone #29, Wuhan 430205, China; xiongjianly@163.com (J.X.); tianfakely@163.com (F.T.)

**Keywords:** MFC, biosensors, toxicity detection, application, environmental monitoring

## Abstract

With the unprecedented deterioration of environmental quality, rapid recognition of toxic compounds is paramount for performing in situ real-time monitoring. Although several analytical techniques based on electrochemistry or biosensors have been developed for the detection of toxic compounds, most of them are time-consuming, inaccurate, or cumbersome for practical applications. More recently, microbial fuel cell (MFC)-based biosensors have drawn increasing interest due to their sustainability and cost-effectiveness, with applications ranging from the monitoring of anaerobic digestion process parameters (VFA) to water quality detection (e.g., COD, BOD). When a MFC runs under correct conditions, the voltage generated is correlated with the amount of a given substrate. Based on this linear relationship, several studies have demonstrated that MFC-based biosensors could detect heavy metals such as copper, chromium, or zinc, as well as organic compounds, including *p*-nitrophenol (PNP), formaldehyde and levofloxacin. Both bacterial consortia and single strains can be used to develop MFC-based biosensors. Biosensors with single strains show several advantages over systems integrating bacterial consortia, such as selectivity and stability. One of the limitations of such sensors is that the detection range usually exceeds the actual pollution level. Therefore, improving their sensitivity is the most important for widespread application. Nonetheless, MFC-based biosensors represent a promising approach towards single pollutant detection.

## 1. Introduction

Fast industrial growth has accelerated environmental pollution globally [1]. Moreover, environmental pollutants are widely distributed and diverse. Among environmental pollutants, heavy metals and organic compounds have attracted particular attention given their large presence in natural environments (soil, air, water, plants, etc.) [2,3]. More recently, according to the U.N. waste monitoring report, it is estimated that approximately 42 million tons of electronic waste is generated globally per annum , mainly composed of heavy metals and organic pollutants [4]. The Greenland MAP Core program has demonstrated organic pollutants in the Arctic show a decreasing trend, except for the polychlorinated biphenyl (PCB) compound group [5]. While the existence of pollutants represents an ecological risk, and also poses a threat to human health and the natural environment, bioremediation processes (e.g., microbial remediation) can remove or degrade heavy metals and organic pollutants. Pollution remediation is inevitably associated with the monitoring of toxic substances in environmental governance. Hence, real-time monitoring of toxicity components in natural environments is of paramount importance.

**Figure 1:**
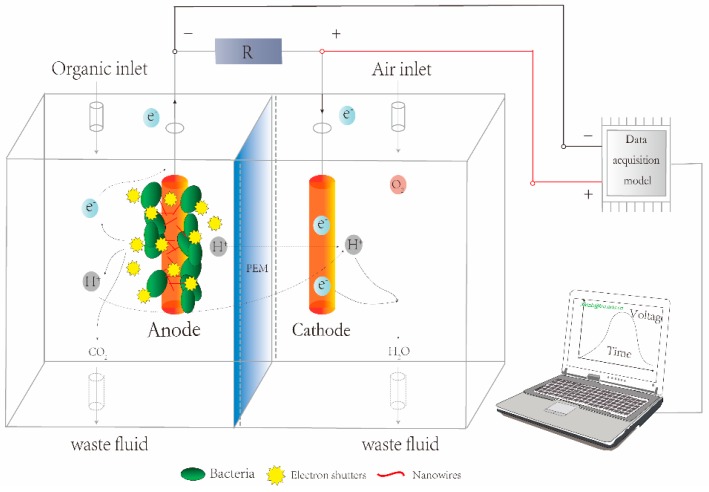
Diagram of a dual chamber microbial fuel cell (MFC).

Fast sensing and analysis of toxic compounds is a great challenge due to their complexity. Traditional toxin detection methods focus on ultraviolet spectrometry and high performance liquid chromatography (HPLC) [6]; however, these analytical methods are usually time-consuming and unsuitable for in situ analysis. Biosensors have been developed as promising tools for fast and selective detection of various analytes [7]. The recognition elements integrated within traditional biosensors, which can be fluorescent molecules, enzymes, or immobilized microorganisms, are costly and require laborious implementation processes [8]. In addition, their low sensitivity and specificity further restricts the potential for large scale applications. Thus, developing a fast and cost-effective biosensor for toxicity detection is extremely urgent. Recently, microbial fuel cell (MFC)-based biosensors have shown great application prospects for environmental pollutant monitoring, since they offer an instant and convenient alternative, ensuring the potential for permanent and long-term monitoring [9,10]. They are usually composed of a cathode chamber and an anode chamber separated by a proton exchange membrane (PEM), allowing protons to migrate from the anode to the cathode and preventing oxygen diffusion into the anodic chamber (Figure 1). Anaerobic respiring bacteria are inoculated into the anodic compartment, where the microbes generate electrons and protons by consuming organic matter. Electrons are conveyed through the anode and pass through an external circuit to the cathode. Combined with the O_2_ from air, protons and electrons react in the cathodic chamber, and eventually form H_2_O.

Previously, MFC-based biosensors have been widely used for water quality testing through monitoring dissolved oxygen (DO), biological oxygen demand (BOD), and chemical oxygen demand (COD). However, these indicators cannot distinguish the dominant organic pollutants [10]. Using MFC-based biosensors for monitoring specific organic compounds may become a novel trend for their application. Although several reviews have focused on the topic of MFC-based biosensors, there is no report on MFC-based biosensors for specific substrates [7,11]. Here, we summarize the latest research outcomes and describes their sensing mechanism. We then further evaluate several factors influencing their behavior and discuss means by which their performances could by improved, more particularly regarding the choices of membrane types and anode materials. In addition, we investigate modified non-linear modelling techniques for MFC-based biosensors, and briefly present possible future research directions, particularly in terms of popularization and potential applications.

## 2. The Mechanisms Governing MFCs Used as Biosensors

**Figure 2:**
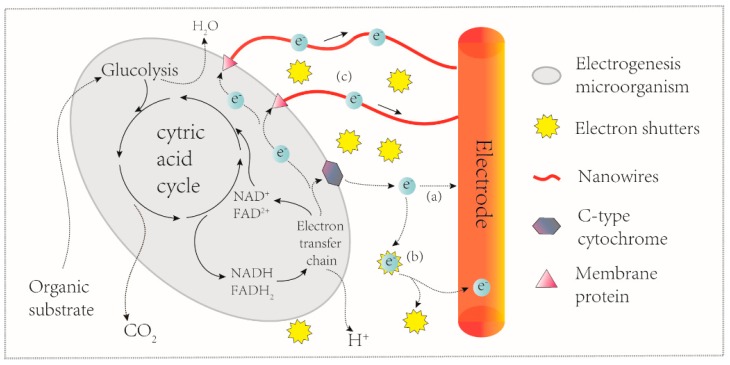
A schematic representation of three microbial extracellular electron transfer mechanisms at anode electrode of MFCs. (**a**) direct transfer via contact and c-type cytochromes; (**b**) indirect electron transfer by electron shuttles; (**c**) direct electron transfer by conductive nanowires.

The electrochemically active microorganisms (EAMs) in an MFC catalyze the degradation of an organic material (fuel), and the electrons subsequently released during this degradation process are transferred to the anode surface [12]. Therefore, the electricity generated by the MFC is the key parameter that directly reflects the metabolic activity of the specific microbes present at the anode. Thus, understanding of the electron generation mechanism of the MFC is important towards comprehending the analytical applications and operating procedures of MFC-based biosensors. *Shewanella oneidensis* MR-1 and *Geobacter sulfurreducens* have often been chosen as representative strains driving the mechanisms of extracellular electron transfer (EET). Based on the available studies, two mechanisms driving charge transfer from biofilms towards the anode surface have been proposed. One is the direct electron transfer (DET) and the other is mediated electron transfer (MET) (Figure 2).

Physical contact between bacterial cell membranes and the MFC anode is a prerequisite of DET. Moreover, the membrane-bound electron transport proteins of EAMs, including c-type cytochromes, multi-heme proteins and OmcZ, can transfer electrons from the inside of the bacterial cell to an outer-membrane (OM) redox protein [13,14]. Some dissimilatory bacteria lack c-cytochromes and instead, use conductive filamentous extracellular appendages termed bacterial nanowires [15,16]. Regarding the MET pathway, flavins and riboflavins secreted by *S. oneidensis* MR-1 have been demonstrated as the electron shutters and dominate the extracellular electron transfer [17,18]. Furthermore, phenazines were also established as intrinsic electron shuttles in *Pseudomonas* species [19]. Although numerous compounds have been introduced into MFCs as exogenous redox mediators to facilitate the electron transfer to electrodes [20,21], these exogenous redox mediators achieved relatively low currents and required continuous addition of the exogenous compound.

As for a microbial biosensor, the current production performance of MFCs can be disturbed by various operational factors, including temperature, pH, salinity, and anode potential [22]. If the MFC functions with non-saturated organic substrates condition, with the abovementioned parameters remaining constant, the biocatalytic activity of electricigens is directly associated with the variations in the concentration of the organic matter fed into the system. The number of electrons transferring to the anode keeps increasing until the concentration of the organic matter reaches a saturation point. This is the basic principle governing the use of MFCs as amperometric sensors for BOD detection in wastewater [23]. In contrast, when using saturated organic substrates, various concentrations of toxic compounds in the input stream can actually inhibit the microbial metabolism activity and substrates consumption, producing changes in the current generated [24].

An inhibition rate (I) has been presented to illustrate the effect of a toxic substance fed into the MFC-based biosensor, which can be calculated using the follow Michaelis-Menten Equation (1):

(1) $$I\left(\text{\%}\right)=\frac{\left|\mathrm{CYnor}-\mathrm{CYtox}\right|}{\mathrm{CY}}\times 100$$

where CY is the Coulombic yield in each peak and it is calculated by integrating the electrical output over time; CYnor and CYtox represent the Coulombic yield in normal wastewater and toxic sample, respectively [25]. In this calculation method, a certain concentration of a toxic pollutant is injected into the anode chamber to observe the Coulombic output, in which three samples are typically utilized as the standard toxicity substrate, including chromium (acute toxin), iron (non-toxic metal) and acetate (organic substrate).

To be applied as a biosensor, the sensitivity of MFC is another significant parameter used to evaluate its functional characteristic. According to Equation (2):

(2) $$sensitivity=\frac{\Delta I}{\Delta c\cdot A}$$

The *sensitivity* of a MFC-based biosensor is defined as the electrical signal change per unit change of analyte concentration. Δ*I* (μA) is the unit change in the current output; Δ*c* (mM) is the unit change in the analyte concentration; and *A* is the electrode surface area (cm^2^) [24].

While the bacterial consortium consumes the organic substrates and consequently releases the electron into anode, the potential difference will be generated between the anode potential and equilibrium redox potential of the substrate [26]. This potential difference is therefore known as the overpotential and its theoretical value can be calculated using the Nernst Equation (3):

(3) $$\eta =E_{an}-E_{0}+\frac{\mathrm{RT}}{nF}\;\ln\frac{\left[\mathrm{ox}\right]}{\left[\mathrm{red}\right]}$$

where η is the overpotential (V); $E_{an}$: the anode potential (V), $E_{0}$: the standard potential of reaction (V); R is gas constant [J (mol·K)^−^^1^]; T, represents temperature (K), *n*, the number of electrons released in the reaction; *F* is Faraday's constant (C mol^−^^1^), and [ox] and [red] (mol L^−1^) are the concentrations of the oxidized and reduced species of the redox couple, respectively [27].

The overpotential disturbance generated by toxic compounds can be correlated to different energy losses at the anode. Under constant conditions, a polarization curve is useful towards evaluating the anode losses and showing the dependence of current on overpotential, combined with enzyme inhibition kinetics, which can be described by the Butler-Volmer-Monod (BVM) Equation (4):

(4) $$I=\mathrm{Imax}\cdot \frac{1-e^{-n\cdot f}}{\beta_{1}\cdot K_{1}\cdot e^{-\left(1-\alpha \right)\cdot n\cdot f}+\beta_{2}\cdot K_{2}\cdot e^{-n\cdot f}+\beta_{3}\cdot \left(Km/S\right)+1}\cdot \beta_{4}$$

In this model, the evaluation of the electric current under fixed overpotential could provide an enhanced sensitivity for a specific toxic compound. In principle*,* by observing the changes in parameters, the effect of four types of enzyme inhibition kinetics can be described, that can help distinguish between various types of toxicity [28]. Although this model cannot deliver a simultaneous estimation of substrate concentration and BVM parameters from current data, by using the weighted least-squares technique to reparametrize the polarization curve, the substrate concentration and consumption rate can be estimated, providing a protocol for on-line detection of toxicity [27].

The EAM enrichment in the anode compartment of a MFC-based biosensor plays an important role, not only as the biocatalyst for current generation from organic substrates, but also as the biological sensing element providing the response signal to various concentrations of toxic compounds. Two strategies have been adopted for the inoculation of EAMs for MFC-based biosensors. In one case, the inoculum source is a compound substance such as anaerobic sludge, soil, or domestic wastewater, which provides a bacterial consortium for the anode chamber [29,30,31]. Alternatively, pure cultures have been used as anode inoculum in recent studies [32,33,34].

Although the analytical performance parameters of MFC-based biosensors, such as detection time, saturation signal, and detection range, show no significant discrepancies when using either a bacterial consortium or specific bacteria as the source of inoculum, pure cultures could maintain high stability and uniformity [35]. Unlike when using a sole bacterial type in the anode chamber, the diversity of a bacterial consortium may vary with different substrates being fed into the system, which could consequently affect the performance of the MFC when used as a biosensor [36]. From another aspect, single bacteria is prone to be manipulated for constructing a more stable and viable toxicant detector. Therefore, employing the single strain as anode biological sensing elements should represent the future research direction towards developing of MFC-based biosensors.

## 3. Analytical Applications of Microbial Fuel Cell-Based Biosensors

A MFC-based biosensor can be defined as an analytical device, integrating bacteria as biological sensing elements to produce a signal proportional to the analyte concentration [37,38]. Compared with conventional biosensors, such as bluegill-, algal- or enzyme-based ones, MFC-based biosensors offer advantages in terms of stability and simplicity, and therefore, have been proposed as promising tools for analytical applications.

### 3.1. MFC as VFA Biosensor

Nowadays, biogas is regarded as a promising renewable alternative energy to replace fossil fuels. However, the unstable anaerobic digestion (AD) process is the main limitation regarding its technological application. To solve the problem, volatile fatty acids (VFAs) are regarded as crucial indicators for monitoring biogas generation [39]. Existing methods for VFA detection, such as high performance liquid chromatography (HPLC), gas chromatography (GC), colorimetric testing and titration, are complex and involve numerous steps [40,41]. Hence, developing a portable VFA determining device is essential for AD process monitoring. In recent years, MFC-based biosensors have been applied for VFA monitoring.

The primary study describing the quantification and analysis of dissolved VFAs was conducted in 2013. Acetate, butyrate and propionate were also discriminated by using Coulombic efficiency and a cyclic voltammetry method. Although the former would require excessive sampling times, a good linear relationship can be observed between the charge and individual VFA species concentration from 5 to 40 mg L^−1^ [42]. Compared to traditional AD, MFC could enhance the degradation rate of propionate and butyrate, indicating a more efficient method for VFA sensing and indeed organic matter removal.

**Figure 3:**
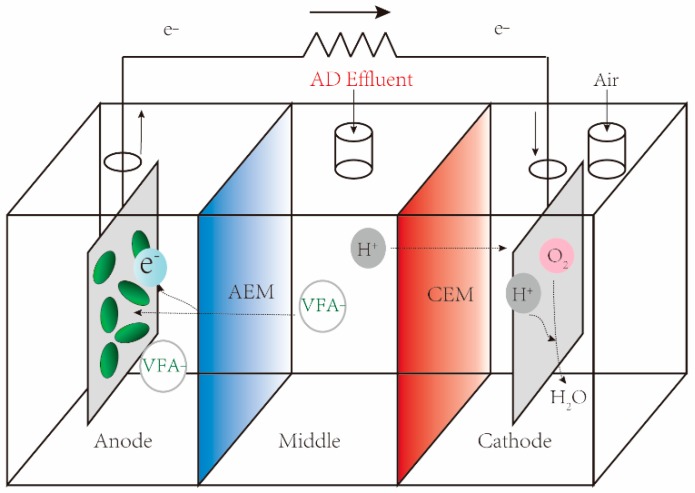
Schematic MFC-based VFA biosensor with three chambers. AEM: anion exchange membrane; CEM: cation exchange membrane.

Based on the principle of microbial desalination cells, Jin et al. [43] proposed a three-chamber VFA monitoring biosensor (Figure 3). In this device, the anaerobic digestion effluent was dosed into the middle chamber and then travelled toward the anode through the AEM, in which the ironized VFAs was utilized by exoelectrogenic microbes for producing electrons. The protons was separated by CEM and combined with the O_2_ to produce water in the cathode chamber. This kind of VFA biosensor showed a broad detection range from 170 mg L^−1^ to 3405 mg L^−1^ due to the separation of bulk solution and anodic microbial community. It also displayed a high selectivity since complex organic matter was retained by AEM which only allowed VFA transport through.

Later on, a microbial electrolysis cell (MEC) was used to facilitate the transportation of VFAs from the cathode compartment to the anode chamber supplemented with an external voltage, thereby shortening the response time. It should be noted this device only required 1 h with a high monitoring concentration of up to 1702 mg L^−1^. Furthermore, the actual performance of this biosensor was further investigated by using real AD effluents and the VFA measurements from the sensor showed no significance differences with those analyzed from GC [44]. The stability and reproducibility of device was achieved without membranes cleaning or replacement after 5 months of operation, demonstrating the robust of this kind of biosensor.

As a matter of fact, a single-chamber MFC (SCMFC) is superior to a dual-chamber MFC by reason of its operability and compactness. Moreover, a study pointed out the SCMFC would be more sensitive [45]. Recently, an air-cathode MFC for online monitoring VFA in anaerobic digesters showed highly sensitive responses of electroactive biofilms with VFAs concentrations increase under four divergent organic wastes. The negative peak of current can be used as an early warning of microbial metabolic inhibition. However, when VFAs increased above 4000 mg L^−1^, electroactive bacteria were subjected to strong inhibition, thus affecting the response current output [46].

To date, MFC-based VFA biosensors show a broad application prospect for monitoring anaerobic digestion process with high sensitivity and comparatively wide response range; however, some issues should be solved in future works, including the effects of fermentation metabolites and other variation of divergent inhibitors. Besides, the behaviors of electroactive biofilms in anodic chamber under different conditions are worth of further investigated. As a result, the onsite operation of MFC-based VFA biosensors needs to be further exploration, especially regarding theirs durability over long term operation.

### 3.2. MFC as BOD Biosensors

Biochemical oxygen demand (BOD) is a crucial parameter used in water quality monitoring, which refers to the amount of dissolved oxygen that microorganisms consume during the oxidation of substances [47]. As a consequence of the significant population expansion and intensifying industrialization and civilization, large quantities of domestic or industrial wastewaters are discharged into rivers, ponds, reservoirs or other surface waters. In most cases, these effluent wastewaters contain very high BOD levels, which can cause severe water quality problems leading to eutrophication, dissolved oxygen depletion, or the death of aquatic organisms [48]. However, conventional methods are not suitable for real-time BOD monitoring, and even require external powered equipment. Thus, a lot of efforts have been directed toward developing MFC-based biosensors. In this section, a brief summary on MFC-based BOD biosensors is provided in Table 1.

**Figure 4:**
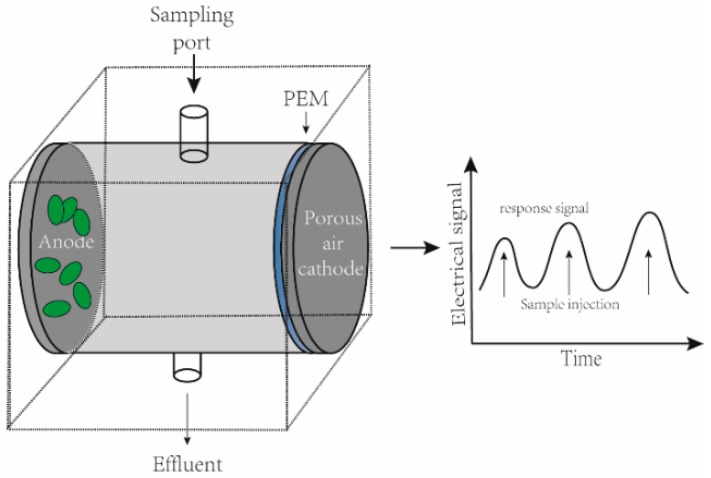
Simplified view of a single-chamber MFC-based BOD biosensor.

The first MFC-based BOD biosensor could only provide an estimated BOD value for industrial wastewater [49]. However, a subsequent study carried out over 5 years confirmed that the MFC has stable performance for BOD monitoring with the limit of detection (LOD) at 2.58 mg L^−1^ [50]. In 2007, Kumlanghan et al. [55] developed a SCMFC for rapid estimating the content of labile organic carbon. In this work, glucose solution was used to simulate the organic matter and the response time was estimated at around 3 to 5 min. The capacity of SCMFC BOD biosensor was also tested by using actual effluent with a very high reproducibility during 7 months of operation [56]. In this study, a smaller reactor (i.e., 12.5 mL) could provide a higher Coulombic efficiency than a larger one (i.e., 25 mL). A simplified diagram of SCMFC based BOD biosensor is shown in Figure 4, in which the anode and cathode were placed in both sides of a cell. The sample was injected from the sampling port and BOD concentration was therefore determined by monitoring the variation of voltage in the MFC.

High redox potential electron acceptors in the anode chamber decrease the signal output obtained from MFCs. Chang et al. [60] demonstrated that respiratory inhibitors (e.g., azide and cyanide) can eliminate the inhibitory effect and significantly improve the MFC performance as a BOD biosensor. In the absence of the electron acceptors, the addition of azide and cyanide did not influence the signal. Besides, the oxygen diffusion into the anode chamber is a serious problem for Coulombic yield, thus affecting the metabolic activity of anaerobic microbes and the sensitivity of BOD biosensors. To correct the defect of this kind of biosensors, a SCMFC, assembled using sulfonated polyether ether ketone (SPEEK), remarkably enhanced the response of this MFC due to its low oxygen permeability. Its sensing range was 62.5% higher than that of Nafion, reaching 650 mg L^−1^ [59].

In situ real-time monitoring of wastewater is meaningful in practical applications, as primary effluents usually contain complicated biodegradable organics and toxic pollutants. An autonomous MFC can be operated for a long time with good characteristics, which indicated the potential for online BOD monitoring [9]. This biosensor is constructed with four MFCs and an energy management system. When the concentration of urine was over an appropriate limit, the sensor could produce a sound and light alarm, lasting for at least 2 days. Similarly, other studies have investigated the possibility of continuously monitoring BOD [53,54]. These results revealed the response current can be proportional to artificial wastewater concentration after a long term hydraulic retention. Peixoto et al. [58] also proposed a submersible MFC (SMFC) for onsite continuous determination of the BOD level of groundwater. This device demonstrated a good stability and its measurable concentration could reach as high as 250 mg L^−1^.

Although almost all BOD biosensors were applied to monitor high BOD values in industrial wastewaters, several studies focused on the determination of low BOD values since the secondary effluents and surface water usually contain low concentrations of organic compounds [54]. In these low BOD biosensors, O_2_-reducing activity at the cathode is considered as a key factor. Kang et al. [51] therefore reported a MFC acting as a low BOD biosensor with a LOD at 5 mg L^−1^ when using a cathode with better affinity for O_2_.

To shorten the response time of the BOD biosensor, the dynamic behavior of MFC was analyzed and optimized. Moon et al. [52] suggested the fuel-feeding rate of MFC should be maintained at 0.53 mL min^−1^, leading to the shortest response time. The experiment results also showed the response time could dramatically reduce from 36 min to 5 min while the anode volume of MFC decreased from 25 mL to 5 mL.

### 3.3. MFC as Toxicants Biosensors

**Figure 5:**
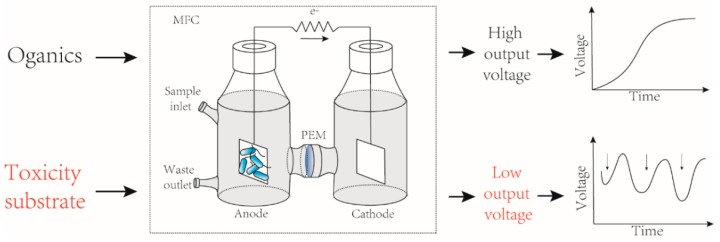
A typical dual-chamber microbial fuel cell used as a toxicity biosensor.

Online monitoring of various toxicants from industrial or domestic wastewaters is a requisite for water resource cyclic utilization and public health safety. Present chemical detection sensors are complicated and involve high operational costs. MFCs can provide a low maintenance and long-term stable solution to this problem. Toxic components can affect the activity of electrogenic microorganisms in biofilms, which contributes to a sudden change (either fall or rise) in the voltage (Figure 5). Depending on the type of substrates being monitored, MFC-based toxicity biosensors could be divided into two main categories i.e., heavy metals biosensors and organic matter biosensors. However, in most cases, the parameters used to establish this classification are ambiguous, since toxin biosensors often display overlapping functions and characteristics.

#### 3.3.1. MFCs as Heavy Metal Biosensors

Heavy metals represent a widely distributed source of pollution, resulting in a series of organs and tissues damage. For example, Hexavalent chromium (Cr^6+^) is a strong carcinogenic substrate. The monitoring of heavy metals through MFCs has grown in recent years. Table 2 provides a review of functional characteristics and analytical performances of MFC-based heavy metal biosensors.

Kim et al. [25] reported that Hg^2+^or Pb^2+^ (1–10 mg L^−1^) could be detected by using a dual-chamber MFC. However, this work only considered limited concentrations of heavy metals. Lately, a MFC was utilized towards monitoring the effects of Cu^2+^ stress on soil microorganisms. The electric signals obtained with glucose-amended soil can be used to evaluate the eco-toxicity of Cu^2+^ with the LOD ranging from 50 to 400 mg L^−1^ [30].

Iron-oxidizing bacterial consortia can be enriched with Fe^2+^ as the sole electron donor [61]. According to this phenomena, Tran et al. [62] therefore constructed a MFC-based Fe^2+^/Mn^2+^ biosensor by inoculating this specific bacterial consortia as anodic electricigens. A linear correlation could be achieved between the current and the Fe^2+^ concentration in the range of 168–1120 mg L^−1^ while the response concentration of Mn^2+^ was less than 165 mg L^−1^. An early Cr^6+^ warning device was also presented, in which *Ochrobactrum anthropi* YC152 was incubated as the anodic microorganism catalyst. The results indicated a stable performance between the concentration of Cr^6+^ ranging from 0.0125 to 0.3 or 0.3 to 5 mg L^−1^ [32].

By using a batch-mode cube MFC, a sensitive shock (sudden change in toxin concentration) biosensor with a reasonable selectivity has been systematically explored. Three heavy metals, including Cr^6+^, Fe^3+^ and NaOAc can be effectively differentiated. The authors also investigated the effect of mixture shock and the results showed the mixed solution with the 8 mg L^−1^ Cr^6+^ and 200 mg L^−1^ NaAc caused a sharp voltage drop within 30 min [63]. In addition, a study has been performed to assess the solitary and joint biotoxicities of heavy metals by employing a single-chamber MFC [64]. The results of binary mixtures of pollutants showed the effect of Cu^2+^ and acephate was antagonistic at 2 mg L^−1^ while was synergistic at 6 and 10 mg L^−1^, and Cd^2+^ and Ni^2+^ were synergistic between 0.2–1.0 mg L^−1^.

The ability to monitor the toxicity of multiple heavy metals could be more practical in application. In 2005, Lee et al. [65] developed a dual MFC system for the monitoring of twelve types of metal. The minimum response concentration of each metal was less than 1.0 mg L^−1^. Later on, using six selected heavy metals, with the LOD at 2 mg L^−1^, to simulate the high or low toxicity, a dual-chamber MFC showed an excellent ability for real-time monitoring toxicity substance [29].

In water clarification, flocculants are widely applied around the world. However, several reports demonstrated that alum can inhibit microorganism activity and event causes nerve poisoning after entering the human body [66,67]. Due to the influence of complex flocs, the in situ evaluation of alum toxicity is quite difficult by using electrochemical methods. A MFC type alum biosensor was therefore designed since the biofilm can be enmeshed inside the flocs [64]. Based on the change of biofilm activity on the electrode, this device could monitor alum concentration range from 0 mg L^−1^ to 500 mg L^−1^.

In general, exoelectrogenic microorganisms in MFCs are heterotrophic, utilizing chemical substrates as their energy resources. Photosynthetic microbial fuel cells (PMFC) represent another strategy by employing the autotrophic microbes as electron donors [68]. They have been applied toward renewable and sustainable electricity production [69]. Recently, Labro et al. [70] proposed a PMFC-based biosensor for monitoring copper, thallium and zinc, in which the electrode surface dwelled in algae and cyanobacteria. This indicates the utility of PMFCs as potential environmental biosensors.

#### 3.3.2. MFCs as Organic Toxin Biosensors

Organic toxins are other common pollution substances found in wastewater, which generally contribute to eutrophication and represent threats to public safety [6]. Table 3 lists the characteristics and performances of organic toxin biosensors employing MFCs.

The toxicity of pesticides such as diazinon and polychlorinated biphenyls (PCBs), has been investigated in an early work by using a dual-chamber MFC [25]. In this study, the detection range of diazinon and PCBs was 1 to 10 mg L^−1^ and 1 to 5 mg L^−1^, respectively. Silicon wafers can be embedded as micro-size electrode elements combined with deep reactive ion etching and standard photolithography. Davila et al. [33] invented a miniaturized MFC as formaldehyde biosensor. This simple and compact apparatus is composed of a proton exchange membrane placed between two silicon plates, further developed into toxicity monitoring equipment. The maximum power density of the micro-fabricated MFC can reach 6.5 μW cm^−2^, which is significantly higher than the maximum power density of 4.4 μW cm^−2^ in a macro-size fuel cell.

Currently, *Shewanella* have been shown as a promising electrogenic bacterium and is extensively used for current generation in MFCs [73,74]. According to the Coulombic response of *S. oneidensis* MR-1 under various toxic substance concentrations, Wang et al. proposed a single-chamber bio-electrochemical systems (BES), and formaldehyde was selected as the typical toxic compound to assess its performance [34]. When 0 mV overpotential was supplied on the anode, the electric response obtained over the concentration range from 100 mg L^−1^ to 1000 mg L^−1^ only requires 2.8 h.

In chemical industry wastewaters, *p*-nitrophenol (PNP) is one of the most commonly found contaminants [75]. The use of physicochemical methods, such as ultraviolet spectrometry, gas chromatography, is unsuitable for in situ real-time monitoring of PNP. Aiming to solve this issue, a specific MFC biosensor for PNP, using *Pseudomonas monteilii* LZU-3, was presented. Moreover, this biosensor showed excellent stability and specificity in regard to the detection of PNP in wastewater containing various additional aromatic compounds (e.g., 2-nitrophenol, 3-nitrophenol, and nitrobenzene) and metal ions (e.g., Fe^2+^, Zn^2+^, Na^+^). The authors of this study also developed a portable device for in situ real-time monitoring and the maximum PNP response concentration could be up to 50 mg L^−1^ [35].

With levofloxacin (LEV) as drug resistance is increasingly occurring, ascribed to the extensive use in the treatment of bacterial infections, a SCMFC was presented for detecting trace LEV concentration [31]. The SCMFC exhibited lasting stability for online monitoring of LEV and its response time only required 5 min.

### 3.4. Comparison of Different Biosensors

MFC-based biosensors have been developed as stable sensing devices to monitor toxins. However, the characteristics of MFC-based biosensors vary with the construction of the MFC, substrates, solution and microbes. For example, the detection limits this kind of biosensors are restricted to toxins (BOD, heavy metal and organic toxins) at the concentrations below 2 mg L^−1^, 0.063 mg L^−1^ and 0.169 mg L^−1^, respectively, which could differ from the actual concentration in the environment [54,70]. In contrast, enzyme-based biosensors can provide a very high specificity for their substrates or inhibitors with detection limits reach 0.003 mg L^−1^ [78], but their application in biosensor construction is restricted by the required tedious and time-consuming enzyme purification. On the other hand, in addition to acting as prosthetic groups of an enzyme, it was well-known that the majority of toxins can distort the protein backbone, leading to enzyme denaturation. Meanwhile, the slow heterogeneous electron transfer from the enzyme to the electrode interface also impedes the wide application of efficient enzymatic biosensors. Microbial biosensors could circumvent the deficiency of enzyme biosensors since the microorganisms encode multiple enzymes in suitable condition and provide a robust reactor. In fact, the LOD of optical microbial biosensors (i.e., bioluminescence and fluorescence biosensors) could reach 0.03 and 0.02 mg L^−1^, respectively, which offer advantages of compactness, flexibility, and a small probe size [79,80].

MFC-based biosensors are considered as a portable and cost-effective detection device for bioactive toxicants comparing with other biosensors. For enzyme-based biosensors, it is essential to maintain a specific environment to avoid enzymatic inactivation. Moreover, the immobilization and purification of enzyme increases the cost of enzyme biosensors and the detection process must rely on specific equipment (e.g., an ultraviolet spectrophotometer), thus is difficult to achieve online monitoring. Likewise, the other types of microbial biosensors need to immobilize the bacteria to the support matrices, and it also complex transducers to achieve the conversion between signal and substrates. On the contrary, the electronic signal output of MFC-based biosensors can directly reflect the toxin concentration.

## 4. The Performance of MFC-Based Biosensors

MFC-based biosensors offer new opportunities for fast monitoring of water quality and food analysis [81,82]. However, the application and performance of MFC-based biosensors is restricted to the detection of analytes at the concentrations below 0.063 mg L^−1^ [54]. Furthermore, the complex substrates present in wastewaters inordinately affect the sensitivity and stability of MFC-based biosensors. Thus, there is an urgent need to address these two limitations of MFC-based biosensors.

### 4.1. Factors That Influence MFC-Based Biosensors

The rate of extracellular electron transfer (EET) is used to characterize MFC-based biosensors’ operation. The anodic biofilm formation efficiency was found to enhance the EET in the absence of mediators. Electrolyte pH affects dramatically the synthesis of riboflavin from *Shewanella*, resulting in the variation of the electrical output for MFCs [18]. Intriguingly, supplementation with riboflavin will decrease the internal resistance and thus reduces the energy loss of the system [83]. However, the use of exogenous mediators might not be applicable to the actual application of MFC, because this external operation may lead to the toxicological problems.

Pretreatment of the carbon mesh has an impact on suitable MFC performance. For example, a carbon mesh treated through the ammonia gas process increased the power to 51 W m^−3^ [84]. Besides, the surface modifications of anode materials represent the most important factors. Ideal anodic materials should have the following features: biocompatibility, conductivity, and chemical stability. The modification of the anode provides a high surface area for the formation of biofilms and increases the power output. Furthermore, the anode type can directly influence the MFC-based biosensor performance; Kong et al. [85] used niobium-doped lanthanum calcium ferrite perovskite as a novel electrode material in MFCs, showing promising results. Some studies of electrode modification also claimed that it can reduce the internal resistance of the system and the start-up time of the reactor [86,87].

In a study focusing on the effects of operating parameters, where a MFC-based biosensor was inoculated with known mixed cultures to determine the BOD concentration, the results showed that methionine, phenylalanine, and ethanol were poor fuels for electricity generation, whereas monosaccharides gave good results [88]. Ji et al. [89] found that electrical signal feedback was more sensitive than pH in the integrated MFC-UASB system, and that limits of sensitivity ranged from 3 × 10^−5^ V (mg L^−1^) ^−1^ to 8 × 10^−5^ V (mg L^−1^) ^−1^ for different concentration ranges. Another study revealed that the type of ion exchange membrane, including cation exchange, anion exchange, monovalent cation exchange, and bipolar membranes, had no significant impact on the sensitivity of MFC-based biosensors [90]. However, the sensitivity is higher at higher overpotential and therefore, at higher current density. Meanwhile, Chen et al. reported that PNP concentration, pH, and temperature influence the performance of PNP biosensors [35]. Hence, in order to achieve a stable baseline current under non-toxic conditions, it is imperative that a MFC-based biosensor should be operated at controlled anode potential, pH and saturated substrate concentrations [91].

### 4.2. Performance Improvement of MFC-Based Biosensors

Although MFC-based biosensors hold great potential as being self-sustainable, without the need for additional signal transducers or external power sources, a change in the concentration of the targeted substrates in the exposed aquatic environmental affects electrogenic microorganisms’ metabolic activities, restricted to the output electrical signal. Thereby, extensive efforts are necessary to improve the capacities of MFC-based biosensors for widespread use.

Improvements in biosensor performance have been achieved with micro-sized MFCs. The miniaturization of biosensor accelerates the cell attachment to the electrodes in anode and then reduces the response time. However, micro-sized MFCs are generally limited as biosensor because of microbubbles interferences in the narrow chamber and its high sensitivity to flow rate variations [92]. When a bubble trap and three electrodes were introduced into the sensing surface, undesirable bubbles can be captured by this trap and thereby provided a stable anodic potential, which enhanced the sensitivity and reliability of this miniature MFC as toxin biosensor [81]. Besides reducing the cost, the miniaturization of MFC can also improve the mass transfer inside the reactor, reducing the difference in concentration of analyte between the input and biofilm, thus leading to a more reliable sensor.

Cathode catalyst is another important factor that influences the performance of MFC-based biosensors. In traditional, the cathode in MFC is usually doped with expensive precious metals (e.g., platinum). A study demonstrated that using FePO_4_ nanoparticles (NPs) as the cathode catalyst instead of Pt/C could improve the sensitivity of MFC-based biosensors. Moreover, this assembled sensor device could dramatically facilitate the voltage output from SCMFC, which provides a powerful guarantee for toxicant detection [31].

Anode chamber is widely used as the sensing element in MFC biosensor; however, the output electric signal of the anodic compartment is easily affected by various parameters. The use of biocathode could greatly reduce the false early warning caused by organic matters or toxicity substrates, which has been extensively applied in MFC for remediation, electric power production and quantification of chemical substrate [93,94]. The amelioration of traditional MFC sensor using biocathode as sensing element achieved a very low detection limit and improved sensitivity for toxicity monitoring [77]. Previous studies demonstrated the hydrodynamic shear rate could restrain production of extracellular polymeric substances and biofilm structures [72]. The work investigated by Shen et al. [71] suggested that under low flow rate with intermittent nitrogen surging could enhance the sensitivity of MFCs as toxicity biosensors.

In MFC biosensors, two flow configurations are employed. One is flow-through and the other hand is flow-by mode. The controlled anode potential (CP) mode delivered better sensitivity than those operated in the constant external resistance (ER) mode over a broad range of anode potentials from −0.41 V to +0.1 V [95]. In addition, anode modifications can improve the performance of a VFA biosensor, and among of six different natural or electroplating polymers tested, poly (pyrrole-alkyl ammonium) resulted in a faster start-up of MFC-based biosensor, while providing improved stability, repeatability and recovery of shorter signal response [96].

### 4.3. Modification of the MFC-Based Biosensors Model

To obtain an accurate signal from a MFC-based biosensor, the overpotential in the anodic chamber should be sustained at a stable baseline. However, various overpotentials could affect the sensitivity of a biosensor. Therefore, it is imperative to investigate the overpotential at which the sensor is most sensitive for the detection of toxicants. Taking consideration of type of toxic matter added to anode compartment, the Butler-Volmer-Monod (BVM) model is also useful to evaluate the influence of overpotential in MFCs [28].

Based on parameter values and data obtained from experimental results carried out under non-toxic conditions, four modified models were applied to fit of the experimental results and the predicted overpotential that contributes to the most sensitive sensor. From this study, the authors verified the overpotential at 250 mV mainly influences the substrate affinity constant (*Km*) and bacterial metabolism. The most sensitive setting for components is at 105 mV overpotential, which affects the ratio of biochemical to electrochemical reaction rate constant (*K1*). When overpotential ranges between 118 mV and 140 mV, the biosensor is sensitive toward toxic component detection and robust against changes in the model parameter *K2* under the simulated conditions [97].

Although mathematical models of MFC-based biosensors have been evaluated [28], there is very little quantitative information about their response peaks. The coefficients (R^2^) between current (cell potential) and oxygen demand (i.e., COD or BOD) have been widely used to assess the performance of MFC-based biosensors; however, it varies greatly. Because this parameter hardly considers the complex relationships between water quality and MFC output, it may provide misleading information. Therefore, there is an urgent need for better MFC output metrics. Feng et al. [98] carried out integrations using two non-linear programming methods, artificial neural networks (ANN), and time series analysis (TSA), to evaluate the performance of MFC-based biosensor. The MFCs generated well-organized, normally-distributed peaks at 150 mg L^−1^ COD or less, while multi-peak signals were obtained at 200 mg L^−1^ COD. ANN predicted the COD concentration accurately with just one layer of hidden neurons, and the TSA model predicted successfully the temporal trends occurring in properly functioning MFCs and in a device that was gradually failing.

## 5. Challenges and Future Prospects

As mentioned before, MFC-based biosensors provide a potential alternative for monitoring diverse toxins. However, there are some critical challenges that limit their practical application, such as the low selectivity and relatively expensive PEM and cathode catalyst. Besides, anaerobic sludge from diverse areas contains different microbial communities. This variation in EAMs introduces a lack of repeatability and is the main limitation of MFC-based biosensors. How to eliminate the above-mentioned challenges and improve the application ability of MFC-based biosensors needs further study.

To improve the current generation and reduce the response time of MFC-based biosensors, the MFCs design needs to focus on decreasing the internal electrical resistance. Screening new anodophilic microbes, microbes groups or consortia with efficient substrate utilization is also important. Very recently, a study demonstrated that one type of bacterium in the consortium can use the electron mediators that are provided by another type of bacterium to transport electrons more efficiently [83]. In addition, the maximum current output from a single MFC could be limited to meet the practical application. By combining the appropriate number of stacked MFC, in theory, we can obtain any desired current or voltage.

Genetically engineered microorganisms based on fusing of receptor and/or reporter proteins to an inducible gene promoter have been widely applied to detect specific toxins. Nonetheless, we still lack effective methods to improve the selectivity of MFC-based biosensors. As the electronic transport mechanism of MFCs is gradually elucidated, it is conceivable to construct genetically engineered bacteria with the ability to reflect the concentration of a given substrate into a voltage output in a MFC biosensor. Besides, many factors can affect the electrogenesis capacity of microorganisms. For example, the extracellular electron mediator (EEM) secreted by bacteria can increase the Coulombic yield of MFCs. Combined with specific receptor proteins of toxins and the EEM regulatory system, it may be possible to fabricate a MFC-based biosensor with high selectivity. In addition, the deletion of key functional genes of EAMs can contribute to improving its substrate specificity, which also is one of promising approaches to enhance the selectivity of MFC-based biosensors.

For easy maintenance and fabrication, MFC-based biosensors should be simplified and portable. Employing modular components and miniaturization design could be useful for the convenient use and mobile operations. Di Lorenzo et al. [24] demonstrated 3D printed devices could provide an effective method for the preliminary design of SCMFC biosensors. From the perspective of economics, noble PEM and cathode catalysts should be instead replaced by other more cost-effective materials. Membrane-less designs are also regarded promising method and ones with the desired output power have been proposed [9].

Although many studies have investigated the performance of MFC-based biosensors in actual effluents, it is essential to explore the sensorial behavior in real contexts since the long-term operation could change the parameters of this system. Furthermore, MFC biosensors must be able to recognize toxic substances in mixed environments and provide a stable output signal. For mixed cultures, understanding the composition and dynamic variations of microbial communities under different substrates is significant, which could reduce perceived risk and accelerate the adoption of this technology.

A number of studies have been carried out to improve the performance of MFC-based biosensors; nevertheless, these works are mainly focused on the one part of the reactor. It must be pointed out that MFC functions as a system, so partial performance may not be directly affected by other parts and an overall strategy should be adopted to design a MFC-based biosensor. We believe that with the current advances in microbial biosensors and progress in modern biotechnology, microbial biosensors will have a promising and bright future.

## 6. Conclusions

This review summarizes the role of MFC-based biosensors in toxic compound detection; MFC-based biosensors have become a potential alternative tool for the rapid monitoring of different substrates, including compounds (VFA) and combined pollutants (e.g., BOD and COD). The substrate concentration under certain conditions has an impact on the formation and activity of biofilms, resulting in current densities proportional to the concentration of pollutants. Furthermore, in MFC-based biosensors, single substrate monitoring is superior to combined pollutant detection, showing excellent selectivity and sensitivity. Therefore, the implementation of MFC as specific substrate biosensor presents an obvious advantage and provides a novel aspect of MFC application.

## Figures and Tables

**Table 1:** MFCs as BOD biosensors.

Source Inoculum	MFC Configuration	Electrode Material	Detection Range (BOD, mg L^−1^)	Saturation Signal	Response Time (min)	Reference
*Clostridium butyricum*	Double chamber	Anode: Pt; cathode: Carbon	10–300	0.120 mA	70	[49]
MFC effluent	Double chamber	Graphite felt	2.58–206.4	1.1 mA ^a^	30–600	[50]
River sediment	Double chamber	Graphite felt	5	ND	180	[51]
MFC effluent	Double-chamber	ND	50–100	1.85 mA ^a^	36	[52]
Activated sludge	Double chamber	Graphite felt	23–200	6 mA ^a^	60	[53]
River sediments	Double chamber	Graphite felt	2–10	6 mA	60	[54]
Activated sludge	Single chamber	Graphite roll	Glucose: 1000–25,000 ^b^	1.6 mv ^a^	60	[55]
Primary wastewater	Single chamber	Carbon cloth	COD: 50–1000 ^b^	0.4 mA	40	[56]
Domestic wastewater	Double chamber	Carbon paper	17–183	222 mA	30	[57]
Underground water	Single chamber	Carbon paper	10–250	233 mA	<40.2	[58]
Activated sludge	Double chamber	Carbon cloth	50–650	0.6 mA ^a^	80	[59]
Neat human urine	Single chamber	Carbon fibre	Urine: 67–813 ^b^	297 mV	69–960	[9]

ND: No data available in original work. ^a^: Estimated using data presented by the authors. ^b^: BOD monitoring capability was demonstrated by using artificial wastewater as the exemplar substrate.

**Table 2:** MFCs as heavy metal biosensors.

Heavy Metals	Source Inoculum	MFC Configuration	Electrode Material	Voltage or Current	Inhibition Ratio	Detection Range (mg L^−1^)	Reference
Hg, Pb	Activated sludge	Double chamber	Carbon felt	0.026–0.040 mA	--	1–10	[25]
Fe, Mn	Iron-oxidizing bacterial consortia	Double chamber	Graphite rod	0.4–0.6 mA 0.1–0.3 mA	--	Fe: 168–1120 Mn: 5.5–165	[62]
KAl(SO_4_)_2_·12H_2_O	MFC effluent	Double chamber	Glassy carbon	6–6.75 A m_2_^−1 a^	--	50–500	[66]
Cu	Soil	Double chamber	Carbon felt	52–354 mV	--	50–400	[30]
Cr, Fe	Fresh wastewater	Single chamber	Carbon felt	53–125 mV	--	Cr: 1–8	[63]
				118–121 mV		Fe: 1–48	
Cr	*Ochrobactrum anthropi* YC152	Double chamber	Plain porous carbon paper	81–258 mV ^a^	--	0.0125–5	[32]
Cu	Domestic wastewater	Single chamber	Carbon felt	--	30–85%	5–7	[71]
Cu, Ni, Cd	Activated sludge	Single chamber	Carbon cloth	--	Cu: 7.5–22.5%	Cu: 1–10	[64]
					Cd: 10–60%	Cd: 0.1–1.0	
					Ni: 3–10% ^a^	Ni: 0.1–1.0	
Cu, Zn	*Paulschulzia pseudovolvox*; Cyanobactera CAWBG64	Double chamber	Carbon cloth	--	Cu: 0–115%	Cu: 0.063–0.189	[70]
					Zn: 0–100% ^b^	Zn: 0.065–0.195	
Cu,Hg Zn, Cd Pb, Cr	Anaerobic sludge	Double chamber	Graphite felts	--	Cu: 7.9–18.48%	Cu: 1–4	[29]
					Hg: 13.99%	Other metals: 0–2	
					Zn: 8,81%		
					Cd: 9.29%		
					Pb: 5.59%		
					Cr: 1.95%		
Cu, Zn Cr, Cd	Anaerobic sludge	Double chamber	Carbon felt	--	Cu: 1.02–9.31%	Cu: 1–25	[72]
					Zn: 0.70–4.16%	Zn: 15–80	
					No data for Cr and Cd	Cr: 0.3–1Cd: 0.4–10	

^a^: Estimated using data presented by the authors. ^b^: Electrogenesis effect.

**Table 3:** MFCs as organic toxin biosensors.

Organic Substrate	Source Inoculum	MFC Configuration	Electrode Material	Voltage or Current	Inhibition Ratio	Detection Range (mg L^−1^)	Reference
Diazinon	Activated sludge	Double chamber	Carbon felt	--	55–61%	1–10	[25]
Polychlorinated biphenyls	Activated sludge	Double chamber	Carbon felt	--	29–38%	1–5	[25]
Acephate	Activated sludge	Single chamber	Carbon cloth	--	8.54–13.34%	1–7	[64]
Glyphosate	Cyanobacteria CAWBG64 *Paulschulzia pseudovolvox*	Double chamber	Carbon cloth	0–125%	--	0.169–0.507	[70]
Formaldehyde	*Geobacter sulfurreducens*	Double chamber	Ti/Ni/Au layer	0–200 mV	--	100	[33]
Formaldehyde	*Shewanella oneidensis* MR-1	Single chamber	Graphite rod	0–200 mV	--	100–1000	[34]
*p*-Nitrophenol	*Pseudomonas monteilii* LZU-3	Double chamber	Carbon felt	115–150 mV	--	50–200	[35]
Formaldehyde	Wild-type *Shewanella oneidensis*	Single chamber	Carbon cloth	0.014–0.023 mA	--	10–100	[76]
Levofloxacin	No Data	Single chamber	Carbon felt	0.41–0.2 mA	--	0.0001–1	[31]
Formaldehyde	MFC effluent	Double chamber	Graphite felt	0.22–0.5 mA	--	5–100	[77]
